# Control of reaching movements by muscle synergy combinations

**DOI:** 10.3389/fncom.2013.00042

**Published:** 2013-04-19

**Authors:** Andrea d'Avella, Francesco Lacquaniti

**Affiliations:** ^1^Laboratory of Neuromotor Physiology, Santa Lucia FoundationRome, Italy; ^2^Center of Space Biomedicine, University of Rome “Tor Vergata”Rome, Italy; ^3^Department of Systems Medicine, University of Rome “Tor Vergata”Rome, Italy

**Keywords:** muscle synergies, reaching movements, human, motor control, intermittency, EMG

## Abstract

Controlling the movement of the arm to achieve a goal, such as reaching for an object, is challenging because it requires coordinating many muscles acting on many joints. The central nervous system (CNS) might simplify the control of reaching by directly mapping initial states and goals into muscle activations through the combination of muscle synergies, coordinated recruitment of groups of muscles with specific activation profiles. Here we review recent results from the analysis of reaching muscle patterns supporting such a control strategy. Muscle patterns for point-to-point movements can be reconstructed by the combination of a small number of time-varying muscle synergies, modulated in amplitude and timing according to movement directions and speeds. Moreover, the modulation and superposition of the synergies identified from point-to-point movements captures the muscle patterns underlying multi-phasic movements, such as reaching through a via-point or to a target whose location changes after movement initiation. Thus, the sequencing of time-varying muscle synergies might implement an intermittent controller which would allow the construction of complex movements from simple building blocks.

## Introduction

We perform reaching movements frequently and effortlessly, for example when eating food or using a tool. Reaching is a prototypical goal directed behavior and, as such, has been investigated extensively in human and non-human primates. Kinematic and kinetic analyses of reaching have revealed invariant features suggesting that the central nervous system (CNS) relies on simple rules for movement planning and execution. For point-to-point movements, hand paths are often roughly straight and tangential velocity is “bell-shaped” (Morasso, 1981). Moreover, paths do not change much with speed (Soechting and Lacquaniti, 1981) or load (Lacquaniti et al., 1982; Atkeson and Hollerbach, 1985). Tangential velocity profiles have the same shape when normalized for speed and distance. Moreover, shoulder and elbow motions can be quasi-linearly related to each other (Soechting and Lacquaniti, 1981; Lacquaniti et al., 1986), as are the corresponding dynamic muscle torques, i.e., the net muscle torque minus the torque required to counteract gravity (Gottlieb et al., 1997).

In contrast, the analysis of the electromyographic (EMG) activity recorded from many muscles acting on the shoulder and elbow joints has revealed complex dependencies of the shape and timing of the EMG waveforms on the movement direction and speed. For reaching in vertical planes, the EMG waveforms are constructed by combining components related to both dynamic and gravitational torques (Flanders, 1991). The waveform components responsible for the dynamic torques (phasic activations) have an intensity and timing that change with the movement direction in a complex manner: each muscle has a distinct spatial and temporal pattern, with a recruitment intensity which is maximal in multiple directions and a recruitment timing that changes gradually across directions (Flanders et al., 1994, 1996). Thus, there is an apparent discrepancy between the kinematic/kinetic regularities of reaching movements and the variability/complexity of the muscle patterns underlying their control.

The control of reaching movements requires a sensorimotor transformation of visual and proprioceptive information about the target and the initial state of the arm into the coordinated activation of many muscles acting on several joints. Because the dynamic relationships between muscle activation and joint torques and between joint torques and joint motions are complex and non-linear, the control of reaching would seem as a challenging task for the CNS (Bernstein, 1967). In robotics, if the geometrical and inertial characteristics of the arm are known or can be estimated precisely, inverse kinematics and inverse dynamics can be used to compute joint angle trajectories and joint torque commands necessary to follow a desired end-effector trajectory. Moreover, if fast sensing and actuation is available, a desired joint angle trajectory can be executed using feedback control. However, it is unlikely that the CNS performs inverse dynamics computations explicitly. Moreover, the CNS has to cope with substantial sensorimotor delays which often make feedback control insufficient. One possibility that has gained increasing support in recent years is that the CNS simplifies the control of goal directed movements by implementing a direct mapping from the initial state of the arm and the goal into appropriate muscle activity patterns through the combination of a few muscle synergies, that is, coordinated recruitments of groups of muscles (Bizzi et al., 2002, 2008; Tresch et al., 2002; Giszter et al., 2007; Ting and McKay, 2007; d'Avella and Pai, 2010; Lacquaniti et al., 2012). Thus, muscle synergies are thought to be stable and reproducible modules organized by the CNS to take the role of “basis functions.” Support for a modular control architecture has been provided in frogs (Tresch et al., 1999; Saltiel et al., 2001; d'Avella et al., 2003; Hart and Giszter, 2004, 2010; Cheung et al., 2005; d'Avella and Bizzi, 2005), cats (Ting and Macpherson, 2005; Torres-Oviedo et al., 2006), monkeys (Overduin et al., 2008, 2012), and humans (Krishnamoorthy et al., 2003; Ivanenko et al., 2004, 2005; d'Avella et al., 2006, 2008, 2011; Torres-Oviedo and Ting, 2007) by identifying a small number of muscle synergies whose combinations explain a large fraction of the variation in the muscle patterns.

Here we first review two notions of muscle synergies commonly used to model the modular organization of muscle patterns, that is, the time-invariant and time-varying muscle synergies. We then review recent results from the analysis of muscle patterns recorded during reaching movements in humans indicating that modulation and superposition of time-varying muscle synergies is a key mechanism for the control of reaching. Time-varying muscle synergies capture spatiotemporal features in the reaching muscle patterns and provide a parsimonious description of the changes of the muscle patterns across conditions, allowing to reconcile the apparent discrepancy between kinematic and kinetic regularities and muscle pattern complexity. Moreover, the superposition and sequencing of time-varying muscle synergies may underlie the intermittent control of complex, multiphasic arm movements.

## Muscle synergies

**Figure 1:**
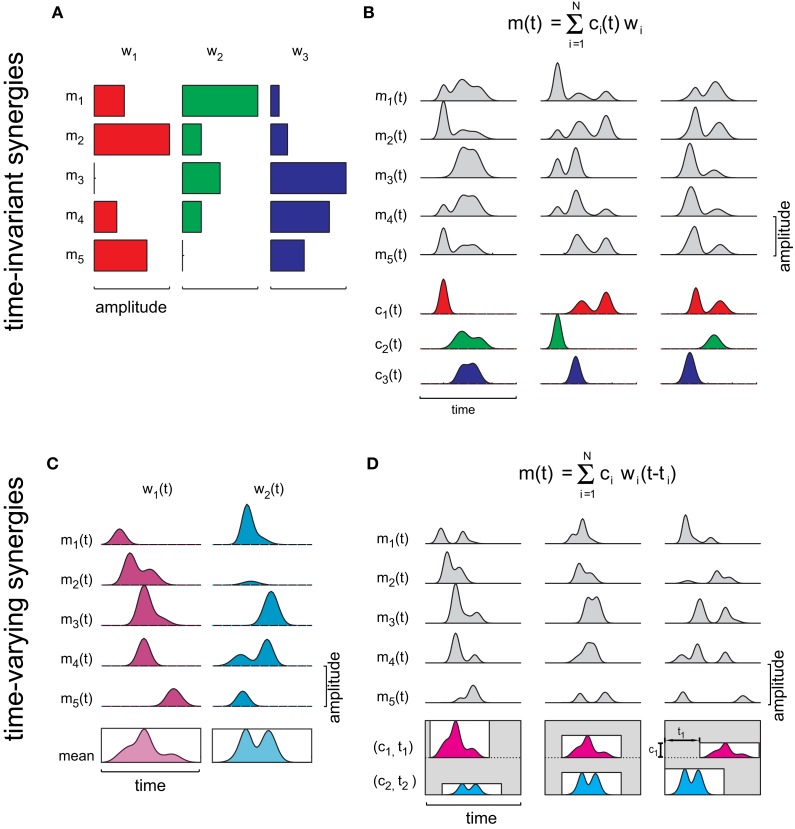
**Concept of time-invariant and time-varying synergies. (A)** Three different activation balances among five muscles are expressed by three vectors (**w**_*i*_), whose components are represented by horizontal bars of different lengths. **(B)** A time-varying muscle pattern [**m**(*t*)] is generated by combining the synergies with time-varying scaling coefficients [*c*_*i*_(*t*)]. Different patterns can be obtained by changing the scaling coefficient waveforms. **(C)** Each one of the two time-varying synergies illustrated is composed by a collection of muscle activation waveforms. The profile inside the rectangle below each synergy represents the mean activation waveform for that synergy. **(D)** A time-varying muscle pattern [**m**(*t*)] is generated by multiplying all waveforms of each synergy by a single scaling coefficient (*c*_*i*_), shifting them in time by a single delay (*t*_*i*_), and summing them together. Different patterns are obtained by changing two scaling coefficients and two delays.

Muscle synergies are building blocks that can be used to control a task in different conditions by selecting a small number of parameters. Synergies are building blocks because they capture a set of features in the muscle patterns that can be reused across movement conditions. In the spatial domain, i.e., across muscles, a muscle synergy captures a specific relationship in the strength of activation of a group of muscles. In the temporal domain, a synergy may capture time-invariant or time-varying relationship among muscles. Considering *D* muscles, a *time-invariant* synergy can be expressed as a *D*-dimensional vector **w** of weighting coefficients specifying the relative activation level of the muscles (Figure 1A). Then, a set of *N* synergies, [**w**_*i*_]_*i* = 1,…,*N*_, can be linearly combined to generate distinct muscle patterns (Figure 1B):

(1) $$m(t)=\sum_{i\;=\;1}^{N}c_{i}(t)w_{i}$$

where **m**(*t*) is a *D*-dimensional vector that specifies the activation of each muscle at time *t* and *c*_*i*_(*t*) is the time-varying combination coefficient for the *i*-th synergy. Across movement conditions, either the synergies **w**_i_ or the activation coefficients *c*_*i*_(*t*), also referred to as temporal components (Ivanenko et al., 2004), may be invariant. A *time-varying synergy*, in contrast, is comprised by a collection of muscle waveforms that can be expressed as a time-varying vector **w**(*t*) (Figure 1C). In this case, the time dependence of the muscle activations is captured by the temporal structure of the synergies and by their onset times (*t*_*i*_) and Equation 1 can be written as (Figure 1D) (d'Avella et al., 2003):

(2) $$m(t)=\sum_{i\;=\;1}^{N}c_{i}w_{i}(t-t_{i})$$

The combination of time-varying synergies can be seen as a special case of anechoic mixture model (Omlor and Giese, 2011). Thus, time-varying synergies provide a parsimonious representation of the motor output because, once the synergies are given, a few scalar amplitude and onset coefficients are sufficient to specify the entire spatiotemporal structure of the muscle pattern. In contrast, with time-invariant synergies the full time-series of combination coefficients must be specified. When both types of synergies are extracted from the same data, the spatial organization of the time-varying synergies, given by the synergy waveforms averaged across time, closely matches the time-invariant synergies (d'Avella and Bizzi, 2005). However, a larger number of time-invariant synergies is required to capture invariant asynchronous activations across muscles (d'Avella et al., 2006).

## Synergies for fast reaching movements

The analysis of the muscle patterns for fast reaching movements in 3D revealed that the complex dependence of the muscle activation waveforms on movement direction results from the combination of 4/5 time-varying synergies (d'Avella et al., 2006). Muscle synergies were identified from the phasic muscle activation waveforms recorded from up to 19 shoulder and arm muscles during fast point-to-point movements between a central location and eight peripheral targets in both a frontal and a sagittal plane. Phasic waveforms are the components of the EMG signal related to accelerating and decelerating the arm and were computed by subtracting the tonic components responsible for balancing gravitational forces and maintaining postural stability. For each subject, an iterative optimization algorithm was used to extract sets of synergies with an increasing number of elements which minimized the average muscle pattern reconstruction error across multiple directions (d'Avella and Tresch, 2002; d'Avella et al., 2003). The number of synergies was determined, as a compromise between model parsimony and reconstruction accuracy, observing the relationship between the amount of data variation explained by the model (R^2^) and the number of synergies. The optimal number of synergies was selected as the number at which the R^2^ curve had a change in slope, suggesting that additional synergies only captured small residual amounts of variation attributable to noise.

**Figure 2:**
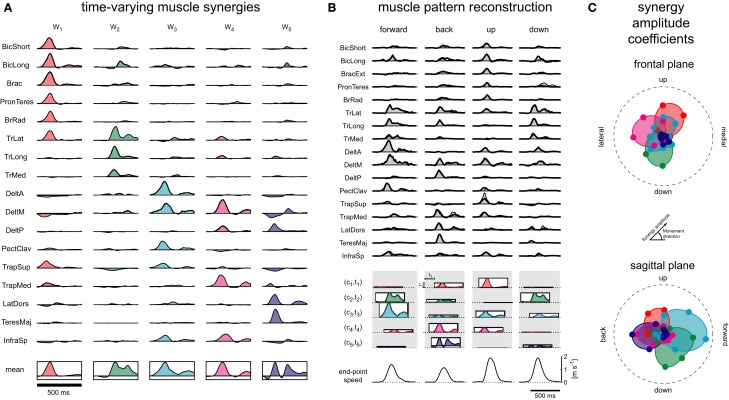
**Muscle synergies for fast reaching movement. (A)** A set of five time-varying synergies, identified from the muscle patterns recorded during point-to-point movements between one central location and 8 peripheral locations in the frontal and sagittal planes with a movement duration below 400 ms. **(B)** The activation waveforms of 17 shoulder and arm muscles for four movement conditions (*columns*) are reconstructed by activating the five synergies with different amplitudes and at different times and then by combining, muscle by muscle, the amplitude-scaled and time-shifted muscle activation waveforms of each synergies. At the *top* of the panel the gray areas represent the averaged EMG activity and the solid black lines the synergy reconstruction. At the *bottom* of the panel, the amplitude scaling coefficient c_*i*_ of each synergy and movement condition is represented by the height of a rectangle and the onset latency *t*_*i*_ and the duration of the synergy is indicated by the horizontal position of the rectangle. The profile within each rectangle represents the mean muscle waveform of each synergy i.e., they are scaled versions of the waveforms shown below each synergy at the *bottom* of panel **A**. **(C)** The amplitude coefficients (c_*i*_) for all five synergies (*color coded*) across all eight movement directions in the frontal (*top*) and sagittal (*bottom*) planes are shown in a polar plot. Thus, for each movement direction, the amplitude coefficient is indicated by the distance from the origin of a colored marker in the corresponding direction. Such polar plots clearly show that the amplitude coefficients are modulated by movement direction (directional tuning) and that each synergy has a specific preferred direction (direction of maximal activation). In most cases the directional tuning is well captured by a cosine function (corresponding to a circle in the polar plot). Adapted from (d'Avella et al., 2006) © 2006 by the Society of Neuroscience, with permission.

Five synergies extracted in one subject (Figure 2A) illustrate the typical basic features. Each synergy recruits a specific subset of muscles with a similar biomechanical action (e.g., elbow flexors in the first synergy, elbow extensors in the second synergy) but each synergy involves muscles involving multiple joints (e.g., brachioradialis and trapezius superior in the first synergy), and the same muscle is often recruited by multiple synergies (e.g., medial deltoid in the third, fourth, and fifth synergy). The synergy waveforms show synchronous bursts of activation in many muscles as well as bi-phasic bursts (e.g., lateral head of triceps in the second synergy) and asynchronous bursts (e.g., long head of biceps in the second synergy). Some muscle waveforms have negative components, indicating an inhibitory drive that reduces the activation of that muscle due to excitatory drive from other synergies or tonic components.

The reconstruction of the muscle patterns by synergy combination for movements in different directions occurs by recruiting the synergies with different amplitude and at different times (Figure 2B). For example, the muscle patterns for a forward movement (*first column*) are generated by recruiting the second plus the third synergy, and the fourth synergy with smaller amplitude and later in time. The second and the third synergies are also recruited in a downward movement (*fourth column*), but with a different balance of activation and a different relative timing. Thus, different muscle patterns underlying reaching movements with different kinematics are captured by selecting a small number of parameters.

Plotting the dependence of the synergy amplitude coefficients on the movement direction in a polar plot (Figure 2C) clearly shows that synergy recruitment depends on movement direction (directional tuning) and that each synergy has a specific direction of maximal activation (preferred direction). In contrast to the dependence of individual muscles (Flanders et al., 1996), in most cases the synergy coefficients have a single peak and, remarkably, the directional tuning is well characterized by a simple cosine tuning (d'Avella et al., 2006). Cosine tuning is characteristic of neural activity in the motor system (Georgopoulos et al., 1982; Caminiti et al., 1991) and represents an optimal encoding of motor commands in terms of accuracy in presence of noise (Todorov, 2002) and minimum effort (Fagg et al., 2002). Thus, the observed cosine tuning of the synergy amplitude coefficients supports the role of muscle synergies as a mechanism for implementing a simple, direct mapping of movement goals into motor commands and suggests that their recruitment may be encoded in motor cortical areas (Overduin et al., 2012).

## Modulation of phasic and tonic synergies with movement direction and speed

If movement direction can be controlled by modulating the recruitment of a few time-varying muscle synergies according to a cosine directional tuning, is movement speed also related to synergy recruitment in a simple way? The invariances observed in the arm kinematics and present in the equations of motions for an articulated arm suggest that a simple scaling rule might be used to control speed. Reaching movements between two given locations are executed at different speeds along an invariant path (Soechting and Lacquaniti, 1981) by scaling in time the entire motion (Atkeson and Hollerbach, 1985). Moreover, the arm motion equations have the property that a solution is invariant for changes in speed (i.e., the resulting joint motion follows the same trajectory with a different time scale) if the dynamic component of the torque profiles is scaled as the inverse of the square of the time scale (Hollerbach and Flash, 1982; Atkeson and Hollerbach, 1985). Thus, the CNS might control the speed of a reaching movement between two locations simply by scaling synergy activation according to movement duration. Such scaling rule would have to be captured by a close-to-quadratic function of the inverse of movement duration (notice, however, that joint torque is related non-linearly to muscle activation).

The analysis of the muscle patterns for reaching in different directions and with different speeds supports the notion of a simple scaling rule for speed control (d'Avella et al., 2008). The patterns recorded during point-to-point movements in eight different directions on the frontal plane with five different movement durations, after scaling in time to equal movement duration, were reconstructed by the combination of three phasic and three tonic time-varying muscle synergies. Phasic synergies, similar in structure to the synergies identified only from the phasic patterns of fast reaching movements and with a similar directional modulation of amplitude and timing coefficients, were also scaled in amplitude by movement speed. The synergy amplitude coefficients for movements in its preferred direction scaled with the maximum speed of the movement according to a power law with an exponent close to two (range over all synergies of five subjects: 1.4–2.7, median 2.0), i.e., approximately in accordance to the torque scaling law. In contrast, tonic synergies, extracted from the muscle pattern without any time-shifts, showed directional modulation in their amplitude coefficients but either non-significant or weak speed dependence (exponent range: 0.1–0.6, median 0.3). Thus, the modulation of a small number of time-varying muscle synergies underlies the control of both direction and speed of point-to-point reaching movements.

## Superposition and modulation of synergies for multi-phasic movements

When reaching a set of different targets in sequence or a target whose location changes after movement initiation, movement kinematics may be complex, with curved paths, and multiple peaks in the tangential velocity. At the kinematic level, such multi-phasic movements can be decomposed as a sequence of superimposed sub-movements, each with the same features of point-to-point movements (Flash and Henis, 1991). As the muscle patterns for point-to-point movements are captured by the combination of a few time-varying muscle synergies, are multi-phasic movements constructed by a sequence of the same point-to-point synergies? If superposition holds at the kinematic level, because of the non-linear dependence of the muscle forces and torques on the arm posture, one expects a simple superposition of muscle patterns and muscle synergies not to hold. However, synergies may provide a simple mechanism for generating the muscle patterns underlying a multi-phasic movement by adjusting a small number of control parameters. To test this hypothesis, the muscle patterns recorded during reaching through a via-point (d'Avella et al., 2006) and to a target changing location after movement initiation (d'Avella et al., 2011) were analyzed using time-varying muscle synergies identified in point-to-point reaching. Indeed, the model of Equation 2 can be extended to allow for the same synergy to be recruited at different, multiple times. When multiple instances of point-to-point synergies were fit to multi-phasic muscle patterns, they reconstructed the muscle patterns with a level of accuracy comparable to that of the point-to-point patterns. However, the recruitment of the synergies, especially those underlying the second phase of the via-point or target change movements, was adjusted with respect to their recruitment in the corresponding point-to-point movement. Indeed, the simple superposition of two, appropriately aligned point-to-point patterns could not reconstruct the multi-phasic patterns with the same accuracy as the synergies. Thus, complex arm movements involving multiple phases appear to be constructed by the modulation and superposition of the same building blocks used for simple point-to-point reaching movements. As time-varying muscle synergies represent an invariant spatiotemporal component of a muscle pattern with a specific duration, the superposition of a set of synergies recruited at different times may be implemented by an intermittent controller.

## Muscle synergies and intermittent control

Sensory feedback is crucial for the control of accurate reaching movements and an internal model of the dynamics of the musculoskeletal system can be exploited to construct an optimal feedback controller (Todorov and Jordan, 2002). However, it might be challenging for the CNS to acquire such a model explicitly and to perform the necessary computations. In contrast, an internal model sufficient for constructing an open-loop controller may be acquired implicitly as a mapping from goals and initial states into motor commands, and feedback might be used for on-line adjustments and trial-to-trial adaptation. Muscle synergies may then provide the basis functions that allow acquiring and using such mapping quickly and efficiently by reducing the number of parameters to be adjusted, stored, and retrieved. An open-loop controller is used before feedback can be processed (Woodworth, 1899; Keele and Posner, 1968), e.g., in the initial phase or for brief movements. However, because of noise and inaccuracy in the model, feedback-driven corrections are required for accuracy. While it is often assumed that such corrections are performed continuously, sensory feedback might also be used intermittently to trigger discrete, open-loop corrections (Doeringer and Hogan, 1998; Gawthrop et al., 2011; Loram et al., 2011). In a synergistic controller, such intermittent corrections may be simply implemented by re-using the mapping of goals and states into synergy recruitment coefficients. Sensory feedback may be processed continuously to update an estimate of the current state and goal, necessary to prepare the synergy coefficients for the appropriate correction. In addition, sensory feedback may be used to construct an error signal which, possibly through a threshold process, triggers a correction by recruiting a set of time-varying synergies. As each synergy has a given duration, different synergies or multiple instances of the same synergy may partially overlap and generate a smooth movement that may appear to be continuously controlled. The fact that the same set of muscle synergies observed in fast point-to-point reaching movements also appear to be recruited in via-point and target-change movements, as reviewed above, supports the notion of a synergy-based intermittent controller.

## Conclusions

Reaching muscle patterns are reconstructed by the combinations of a few time-varying muscle synergies. The complex changes of the activation waveforms of individual muscle across movement direction and speed are captured by the modulation in amplitude and timing of these synergies according to simple rules, such as amplitude cosine tuning for direction and time scaling for speed. Multi-phasic reaching movements, such as reaching through a via-point or toward a target whose location changes after movement initiation, appear to be generated by sequencing and superimposing the same small set of muscle synergies identified in point-to-point movements. Thus, the regularities observed in the muscle patterns across movement conditions suggest that muscle synergies are building blocks used by the CNS to control goal directed movement. However, regularities may derive from optimization or task constraints (Todorov and Jordan, 2002; Tresch and Jarc, 2009; Kutch and Valero-Cuevas, 2012). Direct support for muscle synergies as centrally organized building blocks would come either from identifying their neural substrates or by testing the prediction that motor adaptation must be more difficult if it cannot be achieved recombining existing synergies (d'Avella and Pai, 2010). Recent results in frogs (Hart and Giszter, 2010) and monkeys (Overduin et al., 2012) support a neural organization of muscle synergies both at the spinal and cortical levels. Future investigations of adaptation after novel perturbations of the musculoskeletal system either compatible or incompatible with the synergies will help to clarify whether muscle synergies are merely low-dimensional approximations of the muscle patterns or building blocks organized by the CNS.

### Conflict of interest statement

The authors declare that the research was conducted in the absence of any commercial or financial relationships that could be construed as a potential conflict of interest.
